# Physiology, Taxonomy, and Sulfur Metabolism of the Sulfolobales, an Order of Thermoacidophilic Archaea

**DOI:** 10.3389/fmicb.2021.768283

**Published:** 2021-10-14

**Authors:** Li-Jun Liu, Zhen Jiang, Pei Wang, Ya-Ling Qin, Wen Xu, Yang Wang, Shuang-Jiang Liu, Cheng-Ying Jiang

**Affiliations:** ^1^School of Basic Medical Science, the Xi’an Key Laboratory of Pathogenic Microorganism and Tumor Immunity, Xi’an Medical University, Xi’an, China; ^2^Key Laboratory of Resources Biology and Biotechnology in Western China, Ministry of Education, College of Life Sciences, Northwest University, Xi’an, China; ^3^State Key Laboratory of Microbial Resources, Institute of Microbiology, Chinese Academy of Sciences, Beijing, China; ^4^University of Chinese Academy of Sciences, Beijing, China

**Keywords:** Crenarchaeota, Sulfolobales, taxonomy, sulfur metabolism, sulfur trafficking

## Abstract

The order Sulfolobales (phylum Crenarchaeota) is a group of thermoacidophilic archaea. The first member of the Sulfolobales was discovered in 1972, and current 23 species are validly named under the International Code of Nomenclature of Prokaryotes. The majority of members of the Sulfolobales is obligately or facultatively chemolithoautotrophic. When they grow autotrophically, elemental sulfur or reduced inorganic sulfur compounds are their energy sources. Therefore, sulfur metabolism is the most important physiological characteristic of the Sulfolobales. The functions of some enzymes and proteins involved in sulfur reduction, sulfur oxidation, sulfide oxidation, thiosulfate oxidation, sulfite oxidation, tetrathionate hydrolysis, and sulfur trafficking have been determined. In this review, we describe current knowledge about the physiology, taxonomy, and sulfur metabolism of the Sulfolobales, and note future challenges in this field.

## Introduction

On the basis of analysis of 16S (18S) rRNA gene sequences, Woese proposed in 1977 that archaebacteria are a different group from eubacteria and eukaryotes (Woese and Fox, 1977). In 1990, life on Earth was then divided into three domains: Bacteria, Archaea, and Eukarya. Crenarchaeota, one of the original phyla of the Archaea, mainly comprise sulfur-dependent thermoacidophiles (Woese et al., 1990).

Sulfolobales are an order within the class Thermoprotei, phylum Crenarchaeota, superphylum TACK (Stetter, 1989; Guy and Ettema, 2011). Since the first member of the Sulfolobales was isolated and identified in 1972, only one family, the Sulfolobaceae was constructed, which included nine validly described genera: *Acidianus*, *Metallosphaera*, *Saccharolobus*, *Stygiolobus*, *Sulfodiicoccus*, *Sulfolobus*, *Sulfuracidifex*, *Sulfurisphaera*, and *Sulfurococcus* (Brock et al., 1972; Segerer et al., 1986, 1991; Huber et al., 1989; Karavaĭko et al., 1994; Kurosawa et al., 1998; Sakai and Kurosawa, 2017, 2018; Itoh et al., 2020). Several species of the Sulfolobales have been reassigned to new phylogenetic position according to phylogenetic data and physiological characters during these years, which are detailed below. Members of the Sulfolobales grow autotrophically by oxidizing elemental sulfur (S^0^), hydrogen (H_2_), sulfidic ores, and reduced inorganic sulfur compounds (RISCs), such as thiosulfate. Heterotrophic growth occurs by aerobic respiration, anaerobic sulfur respiration, or by fermentation of organic substrates (Huber and Prangishvili, 2006).

**Figure 1:**
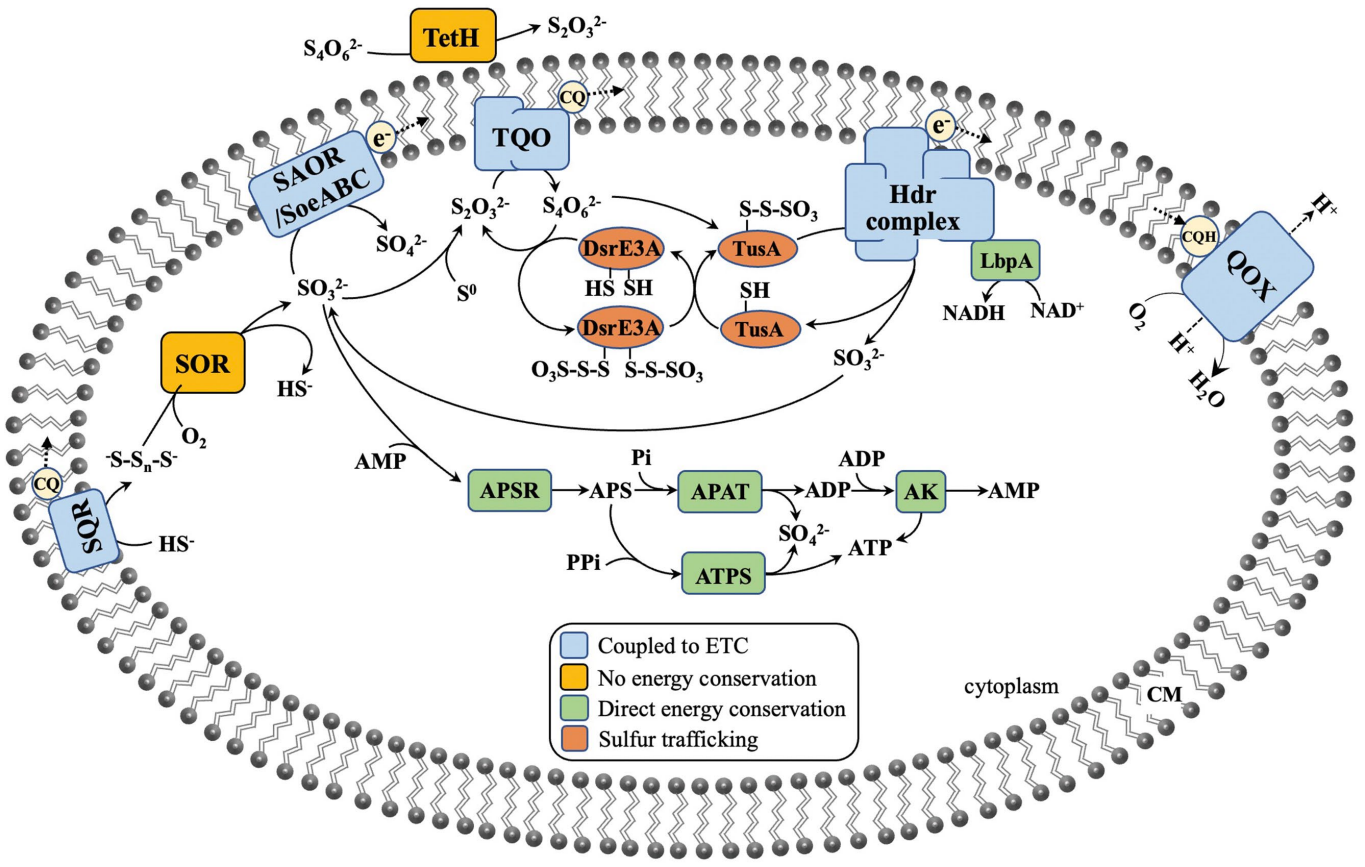
The proposed sulfur oxidation pathway in Sulfolobales. SQR, sulfide:quinone oxidoreductase; SOR, sulfur oxygenase reductase; SAOR, sulfite: acceptor oxidoreductase; SoeABC, sulfite oxidizing enzyme; TetH, tetrathionate hydrolase; TQO, thiosulfate: quinone oxidoreductase; Hdr, heterodisulfide reductase; LbpA, lipoate-binding protein A; Dsr, disulfide reductase; Tus, tRNA 2-thiouridine synthesizing protein; APSR, APS reductase; APAT, adenylylsulfate: phosphate adenylyltransferase; ATPS, ATP sulfurylase; AK, adenylate kinase; CQ, caldariellaquinones; and QOX, quinone oxidoreductase.

The element sulfur exists in various chemical valence ranging from −2 to +6, and RISCs include sulfides (S^2−^, HS^−^, and H_2_S), polysulfide (^−^S-S_n_-S^−^), elemental sulfur (S^0^), sulfite (SO_3_^2−^), thiosulfate (S_2_O_3_^2−^), and tetrathionate (S_4_O_6_^2−^). Because of the diversity of the available forms of sulfur, many enzymes and proteins exist in Sulfolobales for sulfur metabolism, including sulfur-reducing enzymes, sulfur-oxidizing enzymes, sulfur carrier proteins, and sulfur transferases, which cooperate with each other as shown in Figure 1. The sulfur metabolism summarized here contains the reduction of S^0^, the oxidation of RISCs (including sulfide, S^0^, S_2_O_3_^2−^, and SO_3_^2−^), hydrolysis of S_4_O_6_^2−^, and sulfur trafficking. The investigation of functions of these enzymes and proteins in sulfur metabolism is one of the main research aspects regarding the Sulfolobales. Significant research progress has been made over the past decades.

The first complete genome of a member of the Sulfolobales, *Saccharolobus solfataricus* P2, was published in 2001 (She et al., 2001). Now, complete genomic data are available for 69 strains within eight genera (except *Sulfurococcus*) in the order Sulfolobales. From genomic information and enzyme activity analysis, we can better understand the characteristics of sulfur metabolism in these organisms. Here, we summarize the key points to provide a clearer understanding of the taxonomy of Sulfolobales and sulfur oxidation in these organisms.

## Main Features of the Genera in the Order Sulfolobales

Nine genera have been identified in the order Sulfolobales. The first, *Sulfolobus*, was described in 1972 (Brock et al., 1972). In recent years, some species were reclassified to new genera based on morphology, physiology, and phylogenetic evidence. Herein, we summarize the latest progress in taxonomy within the Sulfolobales. The main features of the nine genera and the main species within each genus are described below and in Tables 1 and 2.

**Table 1:** Main characteristics of the nine genera in the Sulfolobales.

Genus name	Cell shape and diameter (μm)	Temp. and pH for growth	DNA G+C content (mol%)	O_2_ requirement	Nutrition type	Autotophic growth-aerobic	Utilization of complex organics	Utilization of sugars
*Sulfolobus*	L/IC, 0.8–1.5	55–95°C pH 1.0–6.5	34–42	Aerobic	Heterotrophic/facultatively chemolithoautotrophic	+	+	+
*Metallosphaera*	L/IC 0.8–1.2	50–80°C pH 1.0–6.5	41–47	Aerobic	Facultatively chemolithoautotrophic	+	+	+/−
*Sulfuracidifex*	IC 1.0–1.8	45–75°C pH 0.4–5.5	38–42	Aerobic	Chemolithoautotrophic/mixtrophic	+	+	+
*Sulfodiicoccus*	IC 0.8–1.5	50–70°C pH 1.4–5.5	52	Aerobic	Heterotrophic	−	+	+
*Acidianus*	C/IC 0.5–2	45–96°C pH 1.0–6.0	30–38	Facultatively anaerobic	Obligately/facultatively chemolithoautotrophic	+	+/−	+/−
*Sulfurisphaera*	C/IC 0.9–1.3	60–96°C pH 1.5–6.0	30–33	Facultatively anaerobic	Facultatively chemolithoautotrophic	+	+	+/−
*Saccarolobus*	IC 0.7–2.2	50–93°C pH 1.5–6.0	31–36	Facultatively anaerobic	Facultatively chemolithoautotrophic	+/−	+	+
*Stygiolobus*	C/IC 0.5–1.8	57–89°C pH 1.0–5.5	38	Obligately anaerobic	Obligately chemolithoautotrophic	−	−	−
*Sulfurococcus*	C	40–80°C	44.6	NA	Facultatively chemolithoautotrophic	+	+	NA

*L, lobed; IC, irregular cocci; C, cocci; +, positive; −, negative; and NA*, original detailed data not available.

**Table 2:** Characteristics of the main members in Sulfolobales.

Species	Cell shape/diameter (μm)	Temp. and pH for growth	DNA G+C content (mol%)	Anaerobic growth (S^0^/Fe^3+^/S_2_O_3_^2−^)	Autotrophic growth-aerobic (S^0^/S_4_O_6_^2−^/pyrite)	Utilization of complex organics	Utilization of sugars	References
**Sulfolobus**								
*Sulfol. acidocaldarius*	L 0.8–1.0	55–80°C (opt. 70–75°C) pH 1.0–5.9 (opt. 2.0–3.0)	36.7	–	Pyrite (w)	Y.E./Pep./Try. /C.A.	D-glucose/starch/sucrose	Brock et al., 1972; Sakai and Kurosawa, 2018
*Sulfol. yangmingensis*	L 0.8–1.5	65–90°C (opt. 80°C) pH 2.0–6.0 (opt. 4.0)	42	ND/−	S^0^/K_2_S_4_O_6_/FeS	Y.E.	D-arabinose/D-glucose/D-galactose/lactose/D-mannose/maltose/raffinose/sucrose	Jan et al., 1999; Sakai and Kurosawa, 2018
*Sulfol. tengchongensis*	IC 1.0–1.2	65–95°C (opt. 85°C) pH 1.7–6.5 (opt. 3.5)	34.4	–	S^0^	Y.E./Try.	D-arabinose/D-fructose/D-galactose/D-xylose/maltose/sucrose	Xiang et al., 2003
**Metallosphaera**								
*M. sedula*	IC 0.8–1.2	50–80°C (opt. 75°C) pH 1.0–4.5 (opt. 2.5)	45	–	S^0^/S_4_O_6_^2−^/pyrite/sphalerite/chalcopyrite	Y.E./Pep./Try./C.A./B.E.	–	Huber et al., 1989; Auernik and Kelly, 2008; Peng et al., 2015
*M. prunae*	IC 1.0	55–80°C (opt. 75°C) pH 1.0–4.5 (ND)	46	–	S^0^/pyrite/sphalerite/chalcopyrite	Y.E./Pep./B.E.	–	Fuchs et al., 1995
*M. hakonensis* (formally *Sulfol. hakonensis*)	L 0.9–1.1	50–80°C (opt. 70°C) pH 1.0–4.0 (opt. 3.0)	46.2	–	S^0^/S_4_O_6_^2−^/FeS/H_2_S	Y.E.	–	Takayanagi et al., 1996; Kurosawa, 2003
*M. cuprina*	IC 0.9–1.0	55–75°C (opt. 65°C) pH 2.5–5.5 (opt. 3.5)	42	–	S^0^/S_4_O_6_^2−^/pyrite	Y.E./Pep./Try./C.A./B.E.	D-glucose/D-xylose/L-arabinose	Liu et al., 2011a,b
*M. tengchongensis*	IC 1.0–1.2	55–75°C (opt. 70°C) pH 1.5–6.5 (opt. 3.5)	41.8	–	S^0^/S_4_O_6_^2−^/pyrite	Y.E./Pep./Try. /C.A./B.E.	–	Peng et al., 2015
**Sulfuracidifex**								
*Sulfura. metallicus* (formally *Sulfol. metallicus*)	C/IC 1.5	50–75°C (opt. 65°C) pH 1.0–4.5 (opt. 2–3)	38	–	S^0^/pyrite/sphalerite/chalcopyrite	Y.E.	Glycogen	Huber and Stetter, 1991; Sakai and Kurosawa, 2018; Itoh et al., 2020
*Sulfura. tepidarius*	IC 1.0–1.8	45–69°C (opt. 65°C) pH 0.4–5.5 (opt. 3.5)	42.4	–	S^0^/FeS/FeS_2_/S_4_O_6_^2−^/chalcopyrite	Y.E./Pep./Try./C.A.	Glucose/maltose/lactose/sucrose/fructose/glycogen/galactose/	Itoh et al., 2020
**Sulfodiicoccus**								
*Sulfod. acidiphilus*	IC 0.8–1.5	50–70°C (opt. 65–70°C) pH 1.4–5.5 (opt. 3.0–3.5)	52	–	–	Y.E./Pep./Try./C.A./B.E.	Arabinose/glucose/xylose/lactose/maltose/sucrose/raffinose/galactose	Sakai and Kurosawa, 2017
**Acidianus**								
*A. infernus*	IC 0.5–2	65–96°C (opt. 90°C) pH 1.0–5.5 (opt. 2.0)	31	S^0^ +H_2_ S^0^ +H_2_S	S^0^	–	–	Segerer et al., 1986; Plumb et al., 2007
*A. brierleyi* (formally *Sulfol. brierleyi*)	IC 1–1.5	45–75°C (opt. 70°C) pH 1.0–6.0 (opt. 1.5–2)	31	S^0^ +H_2_S Fe^3+^ +H_2_S	S^0^/Fe^2+^	Y.E./Pep./Try. /C.A./B.E.	–	Segerer et al., 1986; Plumb et al., 2007
*A. ambivalens* (formally *Desulfurolobus ambivalens*)	IC NA	NA-87°C (opt. 80°C) pH 1.0–3.5 (opt. 2.5)	32.7	S^0^ +H_2_ S^0^ +H_2_S	S^0^	–	–	Plumb et al., 2007
*A. tengchongenses*	C 1.2	55–80°C (opt. 70°C) pH 1.0–5.5 (opt. 2.5)	38	S^0^ +H_2_	S^0^/S_2_O_3_^2−^	–	–	He et al., 2004
*A. manzaensis*	C 0.5–0.8	60–90°C (opt. 80°C) pH 1.0–5.0 (opt. 1.2–1.5)	29.9	Fe^3+^ +S^0^ Fe^3+^ +H_2_	S^0^	Y.E./Pep./Try. /C.A./B.E.	glucose/lactose/mannose/sucrose	Yoshida et al., 2006
*A. sulfidivorans*	IC 0.5–1.5	45–83°C (opt. 74°C) pH 0.35–3.0 (opt. 0.8–1.4)	31.1	S^0^ +H_2_S Fe^3+^ +H_2_S	S^0^/Fe^2+^/pyrite/chalcopyrite/arsenopyrite	Y.E./M.E.	ND	Plumb et al., 2007
**Sulfurisphaera**								
*Sulfuri. ohwakuensis*	C 0.9–1.3	60–91°C (opt. 84°C) pH 1.5–6.0 (opt. 2.0)	32.9	S^0^ +H_2_ Fe^3+^ +Y.E.	S^0^/S_4_O_6_^2−^/pyrite/FeS	Y.E./Pep./Try. /C.A./B.E.	–	Kurosawa et al., 1998; Tsuboi et al., 2018
*Sulfuri. javensis*	IC 0.9–1.3	60–90°C (opt. 80–85°C) pH 2.5–6.0 (opt. 3.5–4.0)	30.6	S^0^ +H_2_ Fe^3+^ +Y.E.	S^0^/S_4_O_6_^2−^/S_2_O_3_^2−^/pyrite/FeS	Y.E./Pep./Try. /C.A./B.E.	–	Tsuboi et al., 2018
*Sulfuri. tokodaii* (formally *Sulfol. tokodaii*)	IC 1.0–1.3	60–96°C (opt. 80°C) pH 1.5–6.0 (opt. 2.5–3.0)	32.8	Fe^3+^ +Y.E.	S^0^/S_4_O_6_^2−^/pyrite/FeS	Y.E./Pep./Try. /C.A./B.E.	D-glucose/D-galactose/D-fructose/lactose/maltose/sucrose/sorbose/raffinose	Suzuki et al., 2002; Tsuboi et al., 2018
**Saccharolobus**								
*Sa. solfataricus* (formally *Sulfol. solfataricus*)	IC 0.8–2.0	50–87°C (opt. 87°C) pH 3.5–5.0 (opt. 4.5)	35.8	Fe^3+^ +Y.E.	–	Y.E./Pep./Try./C.A.	D-arabinose/D-glucose/D-galactose/L-arabinoseD-mannose/lactose/maltose/raffinose/starch/sucrose	Zillig et al., 1980; Sakai and Kurosawa, 2018
*Sa. shibatae* (formally *Sulfol. shibatae*)	IC 0.7–1.5	55–86°C (opt. 81°C) pH 1.5–6.0 (opt. 3.0)	35	Fe^3+^ +Y.E.	Pyrite(w)	Y.E./Pep./Try./C.A.	D-arabinose/D-glucose/D-mannose/lactose/maltose/raffinose/starch/sucrose/L-arabinose	Grogan et al., 1990; Sakai and Kurosawa, 2018
*Sa. caldissimus*	IC 0.8–2.2	65–93°C (opt. 85°C) pH 1.5–6.0 (opt. 3.0)	31.7	Fe^3+^ +Y.E.	Pyrite	Y.E./Pep./Try./C.A.	D-arabinose/D-glucose/D-galactose/D-mannose/lactose/maltose/raffinose/starch/sucrose/L-arabinose	Sakai and Kurosawa, 2018
**Stygiolobus**								
*S. azoricus*	C/IC 0.5–1.8	57–89°C (opt. 80°C) pH 1.0–5.5 (opt. 2.5–3.0)	38	S^0^	–	–	–	Segerer et al., 1991
**Sulfurococcus**								
*Sulfuro. yellowstonii*	C NA	40–80°C NA	44.6	NA	S^0^/Fe^2+^/sulfide minerals	NA	NA	Karavaĭko et al., 1994

*C, cocci; IC, irregular cocci; L, lobed; w, weakly; ND, no data; −, negative; NA, original detailed data not available; Y.E., yeast extract; Pep., peptone; Try., tryptone; C.A., casamino acids; B.E., beef extract; and M.E., meat extract*.

### Sulfolobus

The genus *Sulfolobus* was established in 1972 and is the type genus of the order Sulfolobales. Members of the *Sulfolobus* were first isolated from acid thermal soils and acid hot springs in Yellowstone National Park (United States), El Salvador, Dominica, and Italy (Brock et al., 1972). Eight species of *Sulfolobus* have been characterized, described, and validly named under the International Code of Nomenclature of Prokaryotes (ICNP)^1^; however, six of them were later reassigned to other genera. The type species is *Sulfol. acidocaldarius*, which was isolated from Locomotive Spring in Yellowstone National Park. *Sulfol. yangmingensis* and *Sulfol. tengchongensis* (which is not validly named) were isolated from a geothermal vent in Yang-Ming National Park in northern Taiwan, and an acidic hot spring in Tengchong, Yunnan, China, respectively (Jan et al., 1999; Xiang et al., 2003). Two species, *Sulfol. islandicus* isolated from Icelandic solfataras (Zillig et al., 1993; Reno et al., 2009) and *Sulfol*. sp. A20 isolated from a hot spring in Costa Rica (Dai et al., 2016), were also described and sequenced, although they were not validly named under the ICNP.

Cells of *Sulfolobus* are irregular cocci with frequent lobes, with diameter 0.8–1.5μm. Cells grow in the temperature range 55–95°C (optimal 65–85°C) and pH range 1.0–6.5 (optimal 2.0–4.0). Aerobic and facultatively chemolithoautotrophic growth occurs on S^0^ or a variety of complex organic compounds and sugars. Anaerobic growth of this genus has not been detected (Jan et al., 1999). The type strain *Sulfol. acidocaldarius* cannot oxidize elemental sulfur autotrophically in aerobic conditions (Brock et al., 1972; Huber et al., 1989; Huber and Prangishvili, 2006; Sakai and Kurosawa, 2018). Corresponding to this, genes encoding sulfur oxygenase for sulfur oxidation were not found in its genome (Sakai and Kurosawa, 2018).

### Metallosphaera

*Metallosphaera* contains five members with valid name. The type species *M. sedula* was isolated from a continental solfataric field in Italy (Huber et al., 1989). *Metallosphaera prunae* was isolated from a smoldering slag heap of a uranium mine in Thuringen (Germany; Fuchs et al., 1995), and *M. hakonensis* was isolated from an acidic hot spring at a geothermal area in Hakone (Japan; Takayanagi et al., 1996; Kurosawa, 2003). *Metallosphaera cuprina* (Liu et al., 2011a) and *M. tengchongensis* (Peng et al., 2015) were both isolated from sulfuric hot springs in Tengchong (Yunnan, China). *Metallosphaera hakonensis* originally belonged to *Sulfolobus*, but it was reclassified to *Metallosphaera* in 2003 by Kurosawa (2003) based on phylogenetic evidence, DNA G+C content, and phenotypic properties (Table 2; Takayanagi et al., 1996; Kurosawa, 2003). *Metallosphaera yellowstonensis* was isolated from Yellowstone National Park and introduced in 2011 (Kozubal et al., 2011), which has not been validly named under the ICNP.

Cells of *Metallosphaera* are cocci or irregular cocci with diameter 0.8–1.2μm. Growth happens at 50–80°C (optimal 65–75°C), and pH 1.0–6.5 (optimal 2.5–3.5). *Metallosphaera* are aerobic and facultatively chemolithoautotrophic (Table 1). They can extract metal ions from several kinds of sulfidic ore, such as pyrite, chalcopyrite, and sphalerite. They can also oxidize S^0^ to sulfate but cannot reduce S^0^ (with or without the presence of H_2_). Heterotrophic growth occurs on complex organic compounds, such as beef extract, casamino acids, peptone, tryptone, and yeast extract. *Metallosphaera* cannot use sugars and amino acids (with the exception of *M. cuprina*, which can use a few types of sugar and amino acid, such as D-glucose, D-xylose, L-arabinose, and L-tryptophan; Table 2; Huber et al., 1989; Fuchs et al., 1995; Takayanagi et al., 1996; Liu et al., 2011a; Peng et al., 2015).

### Sulfuracidifex

The genus *Sulfuracidifex* was proposed by Itoh in 2020. The type species is *Sulfura. tepidarius*, which was isolated from a solfataric field at Hakone, Japan (Itoh et al., 2020). Another member is *Sulfura. metallicus*, isolated from continental solfataric fields in Iceland (Huber and Stetter, 1991). *Sulfura. metallicus* was reclassified from *Sulfolobus* because its phenotypic properties and 16S rRNA gene sequences are closer to those of *Sulfura. tepidarius* than to other members of the order Sulfolobales (Itoh et al., 2020).

Cells of *Sulfuracidifex* are irregular cocci with diameter 0.8–1.2μm. Growth occurs at 45–75°C (optimal around 65°C), and at pH 0.4–5.5 (optimal 2.5–3.5). *Sulfuracidifex* is obligate aerobes. Cells grow autotrophically on S^0^, reduced sulfur compounds, or sulfide ores. When grown mixotrophically, *Sulfura. tepidarius* uses several complex organics and sugars, whereas *Sulfura. metallicus* uses only yeast extract or glycogen as carbon sources (Tables 1 and 2; Huber and Stetter, 1991; Itoh et al., 2020).

### Sulfodiicoccus

The type species of genus *Sulfodiicoccus* is *Sulfod. acidiphilus*, isolated from the Hakone Ohwaku-dani hot spring in Japan in 2017 (Sakai and Kurosawa, 2017). At present, it is the only member of the *Sulfodiicoccus*. Growth of the species is significantly inhibited in the presence of S^0^. The DNA G+C content is 52.0mol%, which is remarkably higher than that of the other known species of the Sulfolobales (30.6–46.2mol%; Sakai and Kurosawa, 2017).

Cells of *Sulfodiicoccus* are cocci to irregular cocci with diameter 0.8–1.5μm. Cells grow at 50–70°C (optimal 65–70°C), pH 1.4–5.5 (optimal 3.0–3.5), and 0–2.5% (w/v) NaCl. *Sulfod. acidiphilus* is strictly aerobic and heterotrophic. Growth occurs on various complex substrates or sugars as carbon sources. Chemolithoautotrophic growth does not occur by oxidation of S^0^, pyrite, K_2_S_4_O_6_, Na_2_S_2_O_3_, or FeSO_4_·7H_2_O, or on H_2_ (Sakai and Kurosawa, 2017).

### Acidianus

There are six species described in the genus *Acidianus*: *A. ambivalens* (Zillig et al., 1986; Fuchs et al., 1996), *A. brierleyi* (Segerer et al., 1986), *A. infernus* (Segerer et al., 1986), *A. manzaensis* (Yoshida et al., 2006), *A. sulfidivorans* (Plumb et al., 2007), and *A. tengchongenses*, which is not validly named (He et al., 2004). Among them, *A. infernus* is the type species of *Acidianus* (Segerer et al., 1986). *Acidianus ambivalens* was previously named *Desulfurolobus ambivalens* (Zillig et al., 1986), but, as it is very similar to *A. infernus* in physiological and biochemical features, it was reassigned to the genus *Acidianus* (Fuchs et al., 1996). Members of the *Acidianus* occur in acidic solfataras and marine hydrothermal systems. Cells of *Acidianus* are irregular cocci with diameter 0.5–2.0μm. Cells grow at 45–96°C (optimal 70–90°C), pH 1.0–6.0 (optimal 0.8–2.5), and 0.1–4% (w/v) NaCl. Chemolithoautotrophic growth occurs aerobically by means of S^0^ oxidation or anaerobically *via* S^0^ reduction with H_2_ as electron donor (Tables 1 and 2). *Acidianus infernus* (Segerer et al., 1986), *A. ambivalens* (Zillig et al., 1986), and *A. tengchongenses* (He et al., 2004) are obligately chemolithotrophic. *A. brierleyi* (Segerer et al., 1986), *A. manzaensis* (Yoshida et al., 2006), and *A. sulfidivorans* (Plumb et al., 2007) are facultatively autotrophic and can grow heterotrophically on yeast extract in the absence of S^0^ in aerobic conditions.

### Sulfurisphaera

The genus *Sulfurisphaera* contains three species at present, *Sulfuri. javensis* (Tsuboi et al., 2018), *Sulfuri. ohwakuensis* (Kurosawa et al., 1998), and *Sulfuri. tokodaii* (Suzuki et al., 2002), which were all isolated from acidic hot springs (Kurosawa et al., 1998; Suzuki et al., 2002; Tsuboi et al., 2018). *Sulfuri. tokodaii* formerly belonged to *Sulfolobus* but was reclassified to *Sulfurisphaera* by Tsuboi et al. (2018) based on the latest phylogenetic data (Suzuki et al., 2002; Tsuboi et al., 2018). The type species of this genus is *Sulfuri. ohwakuensis* (Kurosawa et al., 1998).

Cells of *Sulfurisphaera* are irregular cocci with diameter approximately 1μm and grow at 60–96°C (optimal 80–84°C), pH 1.5–6.0 (optimal 2.0–4.0), and 0–1.5% (w/v) NaCl. Cells are facultatively anaerobic. Anaerobic growth occurs on FeCl_3_ in the presence of yeast extract. Chemolithoautotrophic growth occurs on S^0^, S_4_O_6_^2−^, and pyrite in aerobic conditions. The G+C content is in the range 30.6–33.7mol% (Tsuboi et al., 2018).

### Saccharolobus

The type species of the genus is *Sa. solfataricus*, which was first described by Zillig et al. (1980). The other two species are *Sa. shibatae* and *Sa. caldissimus* (Sakai and Kurosawa, 2018). *Sa. solfataricus* and *Sa. shibatae* were originally classified into the genus *Sulfolobus* (Zillig et al., 1980; Grogan et al., 1990). However, later study demonstrated that their abilities to use various sugars were quite different from that of *Sulfol. acidocaldarius*, the type species of *Sulfolobus*. The growth temperature and pH, and facultatively anaerobic characteristics, of *Sa. solfataricus* and *Sa. shibatae* are almost identical to those of *Sa. caldissimus*. Phylogenetic evidence based on 16S rRNA and 23S rRNA gene sequences also helped distinguish *Sa. solfataricus*, *Sa. shibatae*, and *Sa. caldissimus* from *Sulfol. acidocaldarius*. Therefore, *Sa. solfataricus* and *Sa. shibatae* were reclassified as *Saccharolobus* (Sakai and Kurosawa, 2018).

Cells of *Saccharolobus* are irregular cocci. The temperature and pH ranges for growth are 50–93°C and pH 1.5–6.0 (optima 80–85°C and 3.0–4.5), respectively. Cells are facultatively anaerobic, using FeCl_3_ as an electron acceptor and yeast extract as an electron donor. Heterotrophic growth occurs on complex substrates, such as yeast extract and various kinds of sugar. Chemolithoautotrophic growth occurs on pyrite or, poorly, by oxidation of H_2_. S^0^ and K_2_S_4_O_6_ cannot be used as electron donors. The G+C content of this genus is in the range 31.7–35.8mol% (Sakai and Kurosawa, 2018).

### Stygiolobus

*Stygiolobus* isolates were obtained from solfataric fields in the Azores and described by Segerer et al. (1991). The type species is *S. azoricus*, the only member of *Stygiolobus*, which is an obligate anaerobe. Cells are irregular cocci or lobed and are approximately 0.5–1.8μm wide in exponential growth phase, and frequently surrounded by pilus- or fimbria-like appendages. The growth temperature and pH ranges are 57–89°C and 1.0–5.5 (optimum around 80°C and 2.5–3.0), respectively. *Stygiolobus azoricus* is obligately chemolithotroph and grow by means of H_2_–S^0^ lithotrophy. Growth was stimulated by a trace amount of yeast extract (0.005–0.02%; Segerer et al., 1991).

### Sulfurococcus

The genus *Sulfurococcus* contains two species: *Sulfuro. mirabilis* and *Sulfuro. yellowsonensis*. The original characterization manuscripts are both in Russian. However, the abstract (written in English) states that *Sulfuro. yellowsonensis* was isolated from the hydrotherm of Yellowstone National Park (United States) and is a spherical, sulfur-oxidizing thermoacidophile. It grows at 40–80°C. *Sulfuro. yellowsonensis* is a facultative autotroph that grows autotrophically by oxidizing elemental sulfur, ferrous sulfate, and sulfide minerals, and heterotrophically on organic compounds. The DNA G+C content is 44.6mol% (Karavaĭko et al., 1994).

## Shared and Diverse Features of Sulfolobales

All members of the order Sulfolobales are acidothermophiles. Most of them were isolated from terrestrial or aquatic solfatara aeras, which are hot and acidic. They have many phenotypic characteristics in common, but also numerous differences. The phylogenetic relationships of some species were revised in recent years. The main characteristics of the nine genera in the order Sulfolobales are listed in Tables 1 and 2.

### Phenotypic Features

As shown in Table 2, the cells of all Sulfolobales are cocci or irregular cocci, 0.5–2.2μm in diameter. However, they have diverse O_2_ requirements and nutrition types. In general, *Sulfolobus*, *Metallosphaera*, *Sulfuracidifex*, and *Sulfodiicoccus* are obligate aerobes. *Acidianus*, *Sulfurisphaera*, and *Saccharolobus* are facultative anaerobes. *Stygiolobus* is the only obligate anaerobic genus within the Sulfolobales. Most members within Sulfolobales are facultatively chemolithoautotrophic, but *Sulfodiicoccus* species are heterotrophs (Sakai and Kurosawa, 2017). *Stygiolobus* and some species of *Acidianus* are obligately chemolithoautotrophic.

Compared with members of *Sulfolobus*, most *Metallosphaera* have greater ability to oxidize RISCs, such as S^0^, S_4_O_6_^2−^, and sulfidic ores, but lesser ability to use sugars. The members of *Sulfolobus* can use different types of sugar. However, compared with *Sa. solfataricus*, which shows high metabolic versatility and is able to use a broad spectrum of substrates, including mono-, di-, oligo-, and polysaccharides, *Sulfol. acidocaldarius* has a much narrower substrate spectrum. This could be attributed to its relatively small genome, which lacks numerous transport systems for substrate uptake (Lewis et al., 2021).

Both *Sulfurisphaera* and *Saccharolobus* are facultatively anaerobic and facultatively chemolithoautotrophic. They have similar abilities in using FeCl_3_ as an electron acceptor in anaerobic conditions, while their abilities to use RISCs vary. In the case of *Sulfurisphaera*, chemolithoautotrophic growth occurs on various kinds of RISC in aerobic conditions. However, *Saccharolobus* can only oxidize pyrite poorly (Kurosawa et al., 1998; Sakai and Kurosawa, 2018). Although Zillig et al. (1980) described that *Sa. solfataricus* can use S^0^ as an energy source, the data of Sakai and Kurosawa (2018) indicate that this species cannot use pyrite or S^0^ at all (Zillig et al., 1980; Sakai and Kurosawa, 2018).

### Phylogenetic Relationships

**Figure 2:**
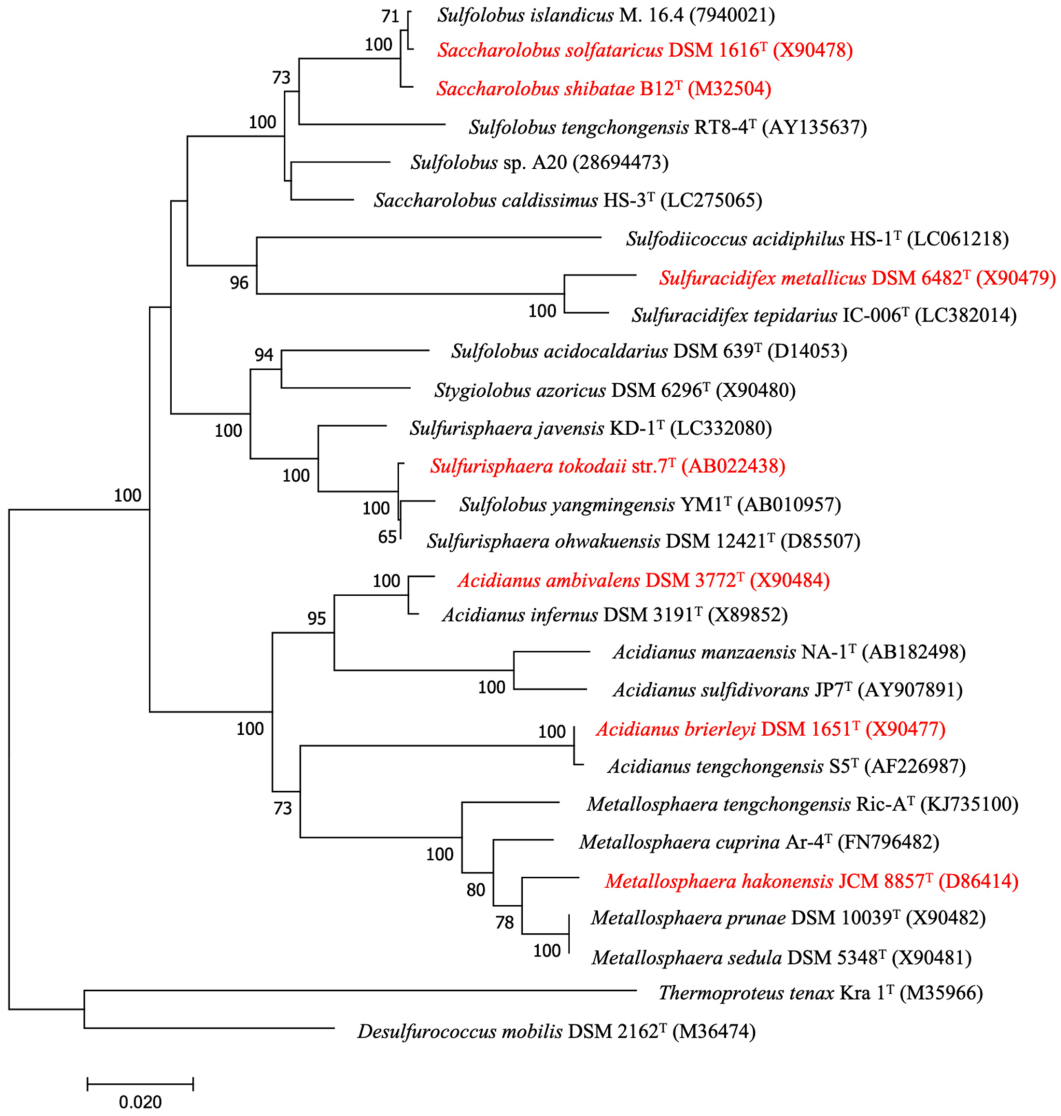
Neighbor-joining phylogenetic tree of the members of Sulfolobales described in this manuscript based on 16S rRNA gene sequences by Mega 7. Numbers at branch nodes represent confidence levels based on 1,000 replicates bootstrap samplings (values greater than 50% are shown), Bar, 0.02 substitutions per nucleotide position. GenBank accession numbers are given in parentheses. The reclassified species are highlighted in red.

**Figure 3:**
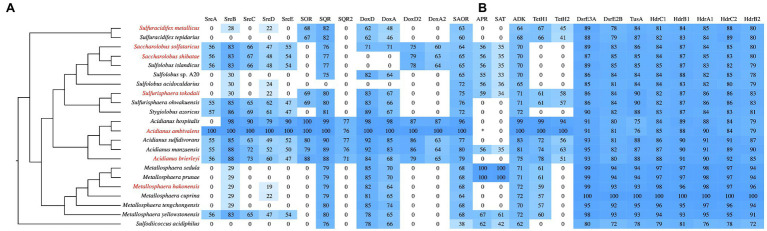
Phylogenetic relationship of the proteins related to sulfur metabolism in the Sulfolobales. **(A)** Phylogenetic tree based on whole-genome sequences constructed by using a Composition Vector (CV) approach (Qi et al., 2004; Zuo and Hao, 2015). K-tuple length: 5. The reclassified species are highlighted in red. **(B)** Protein related to sulfur metabolism within the Sulfolobales and their percentage identity at the amino acid level (blastp, coverage >80%). *Acidianus ambivalens*, *Metallosphaera sedula*, and *Metallosphaera cuprina* proteins are used as queries. Sre, sulfur reductase; SOR, sulfur oxygenase reductase; SQR, sulfide:quinone oxidoreductase; DoxD, thiosulfate:quinone oxidoreductase (TQO) small subunit; DoxA, TQO large subunit; SAOR, sulfite:acceptor oxidoreductase; APR, APS reductase; SAT, ATP sulfurylase; ADK, adenylate kinase; TetH, tetrathionate dehydrogenase; Dsr, disulfide reductase; Tus, tRNA 2-thiouridine synthesizing protein; and Hdr, heterodisulfide reductase. *, the APR activity was detected in *Acidianus ambivalens* (Zimmermann et al., 1999), while no homologous sequence was found when use APR sequence of *Metallosphaera sedula* as query.

With the increasing number of isolates and phylogenetic data, several species of Sulfolobales have been taxonomically reclassified. *Sulfolobus* was the first described genus of the Sulfolobales. Six species that were originally classified as *Sulfolobus* species – *A. brierleyi* (Zillig et al., 1980), *M. hakonensis* (Takayanagi et al., 1996), *Sa. solfataricus* (Zillig et al., 1980), *Sa. shibatae* (Grogan et al., 1990), *Sulfura. metallicus* (Huber and Stetter, 1991), and *Sulfuri. tokodaii* (Suzuki et al., 2002) – have been reclassified into new genera according to later physiological and phylogenetic evidences (Segerer et al., 1986; Kurosawa, 2003; Sakai and Kurosawa, 2018; Tsuboi et al., 2018; Itoh et al., 2020; Table 2; Figures 2, 3).

Although features, such as morphology, temperature and pH for growth, O_2_ requirements, and nutrition types of *Sulfurisphaera*, resemble those of the *Acidianus*, phylogenetic analyses including 16S rRNA gene similarities and DNA–DNA hybridization data distinguish it from the other genera of the Sulfolobales (Segerer et al., 1986; Kurosawa et al., 1998; Tsuboi et al., 2018).

As the phylogenetic tree in Figures 2, 3 show, members of the other genera of Sulfolobales each cluster together or closely, with the exception of the members of *Sulfolobus*, which are dispersed in different clusters. Notably, the 16S rRNA gene sequence of *Sulfol. yangmingensis* is more similar to that of *Sulfuri. tokodaii* and *Sulfuri. ohwakuensis*, and these three species form a clade in the phylogenetic tree (Figure 2). Furthermore, the use of organic compounds and RISCs by *Sulfol. yangmingensis* is also similar to that by *Sulfurisphaera*, although the G+C content of *Sulfol. yangmingensis* (42%) is much higher than that of *Sulfurisphaera* spp. (30–33%; Kurosawa et al., 1998; Jan et al., 1999; Tsuboi et al., 2018). *Sulfol. islandicus*, *Sulfol. tengchongensis*, and *Sulfol*. sp. A20, have been charactered or sequenced but not validly named, are all far from the type strain *Sulfol. acidocaldarius* but related to the clade containing *Saccharolobus*, according to phylogenetic analysis based on 16S rRNA gene sequences and whole genome sequences (Figures 2, 3). Average amino acid identity (AAI) and conserved multi-locus sequence alignment (MLSA) also indicate that *Sulfol. acidocaldarius* is distinct from the other species of *Sulfolobus* (Counts et al., 2021).

In addition, AAI and MLSA data for Sulfolobales suggest that *Sulfod. acidophilus* should be classified into a new family (Counts et al., 2021). All these observations indicate that the phylogenetic positions of the members of Sulfolobales need to be reconsidered.

## Sulfur Metabolism in the Order Sulfolobales

Sulfur metabolism is an important physiological process of many members of Sulfolobales. From genomic information and enzyme activity analysis, several enzymes and proteins related with the metabolism of different RISC have been recognized.

### Reduction of S^0^

Reduction of elemental sulfur is universal among hyperthermophilic archaea. Three genera of Sulfolobales – *Acidianus*, *Sulfurisphaera*, and *Stygiolobus* – reduce S^0^ to H_2_S with H_2_ as the electron donor (Table 2). Two membrane-bound, multisubunit enzymes are involved in S^0^ reduction in *Acidianus*: sulfur reductase (SR) and NiFe hydrogenase. SR is composed of five subunits encoded by the *sreABCDE* gene cluster: a large subunit (SreA), an Fe–S-cluster-containing subunit (SreB), a membrane-anchor subunit (SreC), and SreD and SreE, whose functions are unknown. Both SreA and SreB share sequence similarity with molybdopterin oxidoreductases belonging to the dimethylsulfoxide reductase family. *sreABC* gene clusters are found in *Sulfol. islandicus*, *A. ambivalens*, *A. brierleyi*, *A. manzaensis*, *A. sulfidivorans*, and *Sa. solfataricus*. The NiFe hydrogenase is encoded by an operon with 12 open reading frames, *hynS*–*isp1*–*isp2*–*hynL*–*hynYZ*–*hypDCE*–*hypYZ*–*hoxM*. HynS, HynL, and Isp1 are the small subunit and large subunit of the hydrogenase and the membrane-anchor protein, respectively. HynS and HynL contain [NiFe] and Fe–S clusters, respectively. HypDCE and HoxM are proteins required for hydrogenase maturation. Isp2, HynYZ, and HypYZ are proteins with unknown functions. Electron transfer between NiFe hydrogenase and SR is probably mediated by quinones in *Acidianus* (Laska, 2003).

### Oxidation of RISCs

#### Oxidation of Sulfide

Sulfides (S^2−^, HS^−^, and H_2_S) are widely distributed in soils, ore, wastewater, and marine environments. They are produced partly from mineral deposits, and partly by biological metabolism, including as products of eukaryotic and prokaryotic endogenous catabolism of cysteine and iron–sulfur proteins and dissimilatory metabolism of sulfur-containing inorganic compounds (Kabil and Banerjee, 2010; Lencina et al., 2013; Gao et al., 2017; Bełtowski, 2019). H_2_S is an important electron donor in prokaryotes, such as phototrophic or chemotrophic microorganisms (Reinartz et al., 1998; Sakurai et al., 2010; Klatt et al., 2015). The enzymes involved in maintaining sulfide homeostasis and providing bioenergy in Sulfolobales are sulfide:quinone oxidoreductase (SQR), which are found distributed widely in all domains (they are found in archaea, bacteria, and mitochondria; Hell et al., 2008; Sousa et al., 2018).

SQR homologs are present in all the members of the Sulfolobales listed in Figure 3 except *Sulfol. acidocaldarius*. Only one SQR-encoding gene is present in the genomes of most members of the Sulfolobales. However, *Acidianus* species harbor a second SQR (SQR2), which share 71–77% identities (100% coverage) with SQR from *A. ambivalens* (AaSQR; Figure 3). SQRs are classified into six types based on their structures and sequences (Marcia et al., 2010; Sousa et al., 2018). AaSQR belongs to Type V SQRs (Sousa et al., 2018), which is the first X-ray crystal structure of an archaeal SQR (PDB ID: 3H8L; Brito et al., 2009). AaSQR has one extended capping loop and a cysteine–flavin adenine dinucleotide (FAD) linkage, and contains two monomers in the asymmetric unit (Sousa et al., 2018). It has two redox centers: the covalently bound FAD and a pair of cysteine residues (C178 and C350) bridged by a chain of three sulfur atoms. A channel on the surface of SQR, at the *re*-side of the FAD, is for substrate entry or product exit. The oxidation reaction product, a polysulfide chain (comprising four or five sulfur atoms) or sulfane, is the substrate for SOR. The reduction part of the reaction occurs on the *si*-side of FAD, where the primary electron acceptor, a quinone, is reduced by electrons from sulfide. This process feeds electrons into the respiratory chain and is coupled to energy conservation (Brito et al., 2009). AaSQR is a membrane-anchored protein, most likely facing the cytoplasm (Brito et al., 2009). McSQR (Mcup_0231) from *M. cuprina* Ar-4 was upregulated when cells grew autotrophically on S^0^ compared with growth heterotrophically on yeast extract (Jiang et al., 2014). The ability of McSQR to oxidize sulfide to polysulfide has been shown (data not published by our group).

SQRs are involved in sulfide-dependent energy conservation and in sulfide detoxification to maintain sulfide homeostasis. Microbial oxidation of sulfide is a hot topic in wastewater bioremediation technology and for sulfide removal from soil. Nevertheless, the catalytic mechanism and the function of most SQRs in cells remain to be uncovered.

#### Oxidation of Elemental Sulfur

Elemental sulfur (S^0^), existing mainly in the most stable form, cyclo-S_8_, is insoluble in water (Boulegue, 1978; Suzuki, 1999; Sosa Torres et al., 2020). In Sulfolobales, S^0^ oxidation is catalyzed by SOR, which was first characterized in *A. ambivalens*. SOR catalyzes oxygen-dependent S^0^ disproportionation, with hydrogen sulfide, sulfite, and thiosulfate as the products. Thiosulfate is produced mainly due to the chemical reaction between sulfite and S^0^ (Kletzin, 1989; Kletzin et al., 2004). S^0^ serves both as electron donor and acceptor, and no external cofactors are required by SOR. The reaction is not coupled with energy conservation (Kletzin et al., 2004; Urich et al., 2004).

Genes encoding SOR homologs in Sulfolobales are widespread in all sequenced *Acidianus* species and are also found in *Sulfuri. tokodaii* and *Sulfura. metallicus*. Three SORs within the Sulfolobales have been structurally characterized: AaSOR from *A. ambivalens* (PDB ID: 2CB2; Urich et al., 2004, 2006), AtSOR from *A. tengchongensis* (PDB ID: 3BXV; He et al., 2000; Li et al., 2008), and StSOR from *Sulfuri. tokodaii* (PDB ID: 6M3X, 6M35; Sato et al., 2020).

**Figure 4:**
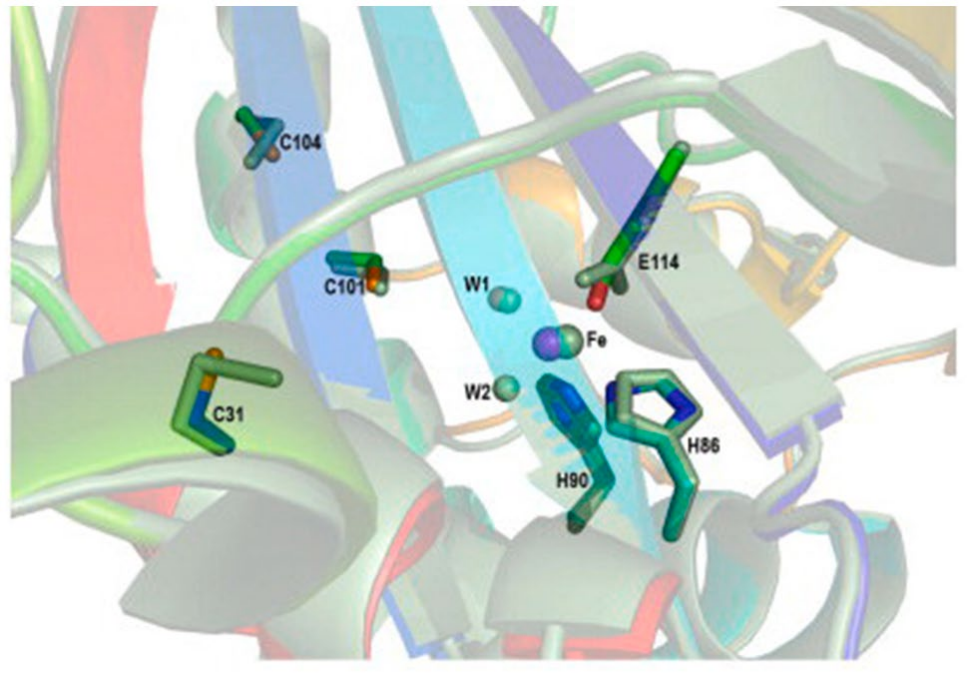
Superposition of the active site between the AtSOR (I432 form) and AaSOR structures. The iron atom is represented as a large sphere and water molecules as small spheres. The residues constituting the active site are shown as sticks. The AaSOR structure is shown in green, while the color of the AtSOR structure is represented by the atom type: yellow, carbon atom; red, oxygen atom (water molecule is included); blue, nitrogen atom; orange, sulfur atom; and magenta, iron atom (Li et al., 2008).

The SORs are homomultimers, each composed of 24 identical subunits, which form a large hollow sphere enclosing a positively charged nanocompartment, where the disproportionation reaction takes place. Six chimney-like protrusions, each composed of four helices that belong to individual monomers, referred to as tetramer channels, are the entry routes of the substrate S^0^; S^0^ enters the tetramer reaction pocket *via* the apolar tetramer channels as a linear polysulfone, rather than as an S_8_ ring. Each monomer possesses an active site pocket comprising a mononuclear non-heme iron site and three conserved cysteine residues (C31, C101, and C104; Figure 4; Urich et al., 2006; Li et al., 2008; Sato et al., 2020). In AaSOR, C31 is involved in binding the substrate S^0^ *via* a cysteine persulfide, and only this residue (among the three cysteines) was essential for the catalytic activity of AaSOR (Urich et al., 2006; Veith et al., 2011). However, the cysteine residues are present as free thiols in AtSOR and StSOR structures. Mutation of any of the three cysteine residues completely abolished the catalytic activity of AtSOR (Li et al., 2008). Mutation of C101 or C104 in StSOR significantly decreased the activity of the enzyme (Sato et al., 2020). The polar reaction products hydrogen sulfide, sulfite, and thiosulfate were proposed to exit the sphere *via* channels located at threefold symmetry axes (Urich et al., 2006; Li et al., 2008; Veith et al., 2011; Sato et al., 2020). SOR activity was detected only in the cytoplasm of *A. ambivalens*, while it is partially located in the cytoplasmic membrane of *A. tengchongensis* (Chen et al., 2005).

SOR is found in all *Acidianus* species and a few other members of the Sulfolobales, such as *Saccharolobus* and *Sulfurisphaera* (Kletzin, 1989; Chen et al., 2007). All archaea harboring SORs have the ability to oxidize S^0^. Notably, although *Metallosphaera* can oxidize S^0^ for growth, no SOR-coding genes are found in their genomes (Figure 3; Auernik et al., 2008; Liu et al., 2011b; Wang et al., 2020). SOR is indicated to be a supplementary but not necessary enzyme for cytoplasmic elemental sulfur oxidation in the sulfur-oxidizing bacteria *Acidithiobacillus* spp. (Wang et al., 2019). Other enzymes may exist that perform S^0^ oxidation in aerobic sulfur-oxidizing Sulfolobales; this requires further research.

#### Oxidation of Thiosulfate

Thiosulfate is further oxidized to tetrathionate by the membrane-bound protein TQO in *A. ambivalens*. *A. ambivalens* TQO consists of two 28-kD DoxD and two 16-kD DoxA subunits, forming an α_2_β_2_ tetramer. TQO oxidizes thiosulfate to tetrathionate with caldariellaquinone (CQ) as the electron acceptor. TQO and the terminal quinol:oxygen oxidoreductase, comprised of two major subunits (DoxB and DoxC) and one minor subunit (DoxE), may form a loose aggregation in the membrane and transfer electrons *via* CQ to reduce O_2_, producing a transmembrane proton gradient for coupled ATP synthesis (Müller et al., 2004).

*doxDA* homologs are found in several genera of Sulfolobales, including *Acidianus*, *Metallosphaera*, *Saccharolobus*, *Sulfodiicoccus*, *Sulfolobus*, *Sulfuracidifex*, and *Sulfurisphaera*. DoxD (Mcup_1713) and DoxA (Mcup_1712) in *M. cuprina* Ar-4 were upregulated when cells grew in autotrophic conditions compared with heterotrophic conditions, as determined by quantitative proteomics (Jiang et al., 2014). Genes encoding DoxD2 and DoxA2, which have low similarity (around 40%) of amino acid sequences to DoxD and DoxA, are present in *Acidianus*, *Saccharolobus*, and *Sulfol. islandicus* (Figure 3). DoxD2 and DoxA2 are separated from DoxDA phylogenetically, and their functions are still unclear (Müller et al., 2004).

#### Oxidation of Sulfite

There are two pathways of oxidation of sulfite to sulfate: direct and indirect oxidation. The direct oxidation of sulfite to sulfate in *A. ambivalens* is catalyzed by sulfite:acceptor oxidoreductase (SAOR), a membrane-bound molybdenum protein. The electrons from sulfite oxidation are probably transferred to CQ, feeding into the respiratory chain. Genes encoding SAOR homologs are found in all the sequenced Sulfolobales (Figure 3). The sulfite produced during sulfur metabolism is in the cytoplasm, while sulfate produced by the membrane-bound SAOR is released to the outside of the cell. However, it is still unknown whether SAOR transports sulfate across the membrane, or if a sulfate transporter is present (Zimmermann et al., 1999). It was reported that sulfite was readily oxidized to sulfate through the direct pathway in the purple sulfur bacterium *Allochromatium vinosum*, catalyzed by the heterotrimeric membrane-bound sulfite-oxidizing enzyme complex SoeABC (Dahl et al., 2013). The sequences of SoeABC subunits were detected in all *Metallosphaera* species. Whether this direct sulfite oxidation pathway works in sulfur-oxidizing archaea remains to be established (Wang et al., 2020).

The indirect sulfite oxidation pathway is catalyzed by adenylylsulfate or adenosine 5'-phosphosulfate (APS) reductase and ATP sulfurylase (also named ATP:sulfate adenylyltransferase, encoded by the *sat* gene) or adenylylsulfate:phosphate adenylyltransferase (APAT, formerly named ADP sulfurylase). APS is an intermediate, involved in substrate-level phosphorylation. In the first reaction, APS reductase catalyzes APS formation from sulfite and AMP, and releases two electrons. The APS can be used in two ways: One is reacting with pyrophosphate (Ppi) catalyzed by ATP sulfurylase, forming ATP and sulfate; the other is in production of ADP and sulfate catalyzed by APAT in the presence of phosphate (Pi). ADP is then converted to ATP by adenylate kinase (Kappler and Dahl, 2001). The activities of APS reductase, APAT, and adenylate kinase were detected in the cytoplasm in *Acidianus ambivalens*, revealing indirect oxidation of sulfite *via* the APS and ADP pathway (Zimmermann et al., 1999). According to our BLAST search results, genes encoding APS reductase and ATP sulfurylase are also present in *A. manzaensis*, *M. sedula*, *M. yellowstonensis*, *Sulfuri. tokodaii*, *Sulfod. acidophilus*, *Saccharolobus*, and *Sulfolobus* species, indicating indirect sulfite oxidation occurs in these organisms, probably *via* APS to form ATP and sulfate (Figure 3), although biochemical evidence for this is still lacking. Neither APS reductase- nor ATP sulfurylase-encoding genes are found in *Acidianus species*, *M. cuprina*, *M. hakonensis*, *Sulfuri. ohwakuensis*, and *Sulfuracidifex* species (Figure 3). The indirect sulfite oxidation pathways in these organisms are still unclear.

### Hydrolysis of Tetrathionate

Tetrathionate, the product of TQO, is further hydrolyzed by tetrathionate hydrolase (TetH), a pseudoperiplasmic protein attached to the S-layer, with an overall β-propeller structure. In *A. ambivalens*, TetH was found only in cells grown on tetrathionate; the gene is poorly expressed in cells grown on sulfur (Protze et al., 2011). TetH secreted by *A. hospitalis* YS8 forms zipper-like particles (ZLPs). The amounts of ZLPs that increased after cells were treated by mitomycin C, UV light, or by freezing in liquid nitrogen and rapid thawing, and they decreased to nondetectable levels after cells adapted to their growth conditions. TetH from *A. hospitalis* YS8 has 99% identity with that from *A. ambivalens*; both are stimulated by general stress (Krupovic et al., 2012).

TetH-coding genes exist in strictly or facultatively chemolithoautotrophic members of the Sulfolobales, which can grow in tetrathionate (Figure 3). Two copies of TetH-encoding genes (*tetH1* and *tetH2*) are found in *Acidianus*, *Sulfuracidifex*, and *Sulfurisphaera* species. TetH1 and TetH2, the function of which is unknown, cluster in distinct clades in a dendrogram (Protze et al., 2011).

### Heterodisulfide Reductase

Heterodisulfide reductase (Hdr) is an iron–sulfur protein first discovered in methanogenic archaea that catalyzes reversible reduction of the heterodisulfide (CoM–S–S–CoB) of the thiol-coenzymes M (CoM–SH) and B (CoB–SH), coupled with energy conservation. Hdr is composed of three subunits, HdrA, HdrB, and HdrC. HdrA contains a typical FAD-binding motif and four [4Fe–4S] cluster-binding motifs. HdrB harbors two similar non-cubane [4Fe–4S] clusters and each cluster consist of fused [3Fe–4S]-[2Fe–2S] subcluster sharing one iron and one sulfur. The ferredoxin-like HdrC contains two [4Fe–4S] cluster-binding motifs (Hedderich et al., 2005; Wagner et al., 2017). Hdr complex-like proteins in sulfur-oxidizing bacteria and archaea are encoded by the gene cluster *hdrC1B1A-hyp-hdrC2B2* (Liu et al., 2014). The Hdr complex in the thermophilic bacterium *Aquifex aeolicus* is a membrane-bound protein composed of at least five subunits: HdrA, HdrB1, HdrB2, HdrC1, and HdrC2 (Boughanemi et al., 2016). The Hdr complex is supposed to oxidize disulfide intermediates to sulfite and deliver the collected electrons to the membrane quinol pool. Furthermore, sulfur trafficking proteins, such as TusA and DsrE, are involved in transferring the sulfur groups to Hdr (Quatrini et al., 2009). Recent evidence showed that the Hdr complex oxidized thiosulfate to sulfite in *Hyphomicrobium denitrificans*, and the electrons produced may be transferred *via* a lipoate-binding protein (LbpA) to generate NADH (Cao et al., 2018; Koch and Dahl, 2018). The expression of Hdr subunits in *M. cuprina* Ar-4 increases when cells are grown in autotrophic conditions compared with heterotrophic conditions, indicating the participation of the Hdr-like complex in sulfur oxidation in *M. cuprina* (Jiang et al., 2014).

### Sulfur Trafficking

Sulfur trafficking is normally required for delivery of sulfur-containing groups as protein-bound forms to the sulfur-catalyzing enzymes. During this process, the unstable sulfur groups can be protected. The active site of TQO is suggested to face the cytoplasm; the tetrathionate produced by thiosulfate oxidation is thus released to the cytoplasm. However, tetrathionate is unstable at the near-neutral pH in the cytoplasm (Protze et al., 2011). Whereas the (*rhd*–)*tusA*–*dsrE2* gene cluster is widely distributed in phototrophic and chemotrophic sulfur-oxidizing bacteria for transfer of sulfane sulfur, the *dsrE3A*–*tusA*–*hdr* gene cluster is ubiquitous in Sulfolobales (Figure 3). TusA appears to be a central and common protein for sulfur trafficking in sulfur-oxidizing pathways (Dahl, 2015). It has been proven in *M. cuprina* that the *dsrE3A*–*tusA*–*hdr* gene cluster is important in trafficking the sulfane sulfur of tetrathionate to prevent its biological toxicity. As shown in Figure 1, DsrE3A and TusA can both react with tetrathionate to form protein–Cys–S–thiosulfonate, which is stable in the cytoplasm. Then, DsrE3A–Cys–S–thiosulfonate transfers one thiosulfonate to TusA, forming TusA–Cys–S–thiosulfonate, and releases another thiosulfonate to TQO. The reverse transfer reaction does not happen. Next, the thiosulfonate combined with TusA serves as the substrate of the Hdr-like complex to produce sulfite for SAOR/SoeABC. The sulfane group remaining on TusA is then oxidized and released (Liu et al., 2014; Dahl, 2015).

## Conclusion

The order Sulfolobales, phylum Crenarchaeota, is distributed in acidic and hot terrestrial or aquatic solfatara aeras and includes nine validly named genera. On the basis of new physiological data and phylogenetic analysis, several species have been reassigned to new taxa over the years. Furthermore, *Sulfol. yangmingensis* should be reclassified in genus *Sulfurisphaera*. *Sulfol. islandicus*, *Sulfol. tengchongensis*, *Sulfol*. sp. A20, and some other *Sulfol*. sp. strains might be placed in the genus *Saccharolobus* according to phylogenetic analysis. Moreover, it is proposed that *Sulfod. acidiphilus* should be used as the type strain of a new family. More newly isolates and their physiological and phylogenetic data are needed to support the reclassification.

Sulfolobales possess a broad array of physiological traits, such as a pH range for growth of 0.4–6.5, a temperature range for growth from 45 to 96°C, different O_2_ requirements (including obligate aerobes, facultative aerobes, and obligate anaerobes), different nutrition types (including heterotrophs, mixotrophs, and chemolithoautotrophs), and DNA G+C content from 30 to 52mol% (Tables 1 and 2). Most Sulfolobales are sulfur or RISC oxidizers or reducers, and they are considered to play important roles in the sulfur cycle of Earth. Some proteins and enzymes involved in sulfur metabolism have been characterized (Figure 1). It seems no universal pathway exists, and the proteins involved in sulfur metabolism vary in different species (Figure 3). Gaps remain in the sulfur metabolism pathways of Sulfolobales: (i) How does element sulfur access to the cytoplasm or do cytomembrane proteins exist to directly oxidize element sulfur? (ii) Which enzyme catalyzes S^0^ oxidation in the species without SOR? (iii) What are the functions of DoxD2 and DoxA2? (iv) How does sulfate transport across the membrane? Further research is required in the above area for understanding these questions.

## Author Contributions

S-JL and C-YJ modified and edited the manuscript. ZJ constructed the phylogenetic tree. PW and Y-LQ provided the information of SQR, SAOR, and TetH. WX and YW contributed to the final version of the manuscript. L-JL wrote the manuscript. All authors contributed to the article and approved the submitted version.

## Funding

This work was funded by the National Natural Science Foundation of China (grants no. 91851206, 31600040, and 31670124), the fellowship of China Postdoctoral Science Foundation (2021M692614), the Key Research Program of Chinese Academy of Sciences (ZDRW-ZS-2018-1), the Joint Funds of Innovation Academy for Green Manufacture, Chinese Academy of Sciences (IAGM2020C24), the CAS Engineering Laboratory for Advanced Microbial Technology of Agriculture, Chinese Academy of Sciences (KFJ-PTXM-016), and the Supporting Foundation of Xi’an Medical University (grants nos. 2017PT29 and 2017PT40).

## Conflict of Interest

The authors declare that the research was conducted in the absence of any commercial or financial relationships that could be construed as a potential conflict of interest.

## Publisher’s Note

All claims expressed in this article are solely those of the authors and do not necessarily represent those of their affiliated organizations, or those of the publisher, the editors and the reviewers. Any product that may be evaluated in this article, or claim that may be made by its manufacturer, is not guaranteed or endorsed by the publisher.

## Footnotes

^1^ https://lpsn.dsmz.de/genus/sulfolobus
